# Thresholds of skin sensitivity are partially influenced by mechanical properties of the skin on the foot sole

**DOI:** 10.14814/phy2.12425

**Published:** 2015-06-09

**Authors:** Nicholas D J Strzalkowski, John J Triano, Chris K Lam, Cale A Templeton, Leah R Bent

**Affiliations:** 1 University of GuelphGuelph, Ontario, Canada; 2 Canadian Memorial Chiropractic CollegeToronto, Ontario, Canada

**Keywords:** Epidermis, foot sole, mechanical properties, monofilament, vibration

## Abstract

Across the foot sole, there are vibration and monofilament sensory differences despite an alleged even distribution of cutaneous afferents. Mechanical property differences across foot sole sites have been proposed to account for these differences. Vibration (VPT; 3 Hz, 40 Hz, 250 Hz), and monofilament (MF) perception threshold measurements were compared with skin hardness, epidermal thickness, and stretch response across five foot sole locations in young healthy adults (*n* = 22). Perceptual thresholds were expected to correlate with all mechanical property measurements to help address sensitivity differences between sites. Following this hypothesis, the MedArch was consistently found to be the thinnest and softest site and demonstrated the greatest sensitivity. Conversely, the Heel was found to be the thickest and hardest site, and was relatively insensitive across perceptual tests. Site differences were not observed for epidermal stretch response measures. Despite an apparent trend of elevated sensory threshold at harder and thicker sites, significant correlations between sensitivity measures and skin mechanical properties were not observed. Skin hardness and epidermal thickness appeared to have a negligible influence on VPT and minor influence on MF within this young healthy population. When normalized (% greater or smaller than subject mean) to the subject mean for each variable, significant positive correlations were observed between MF and skin hardness (*R*^2^ = 0.422, *P* < 0.0001) and epidermal thickness (*R*^2^ = 0.433, *P* < 0.0001) providing evidence that skin mechanics can influence MF threshold. In young healthy adults, differences in sensitivity are present across the foot sole, but cannot solely be accounted for by differences in the mechanical properties of the skin.

## Introduction

Cutaneous feedback from the soles of the feet plays an important role in the control of gait and standing balance. When foot sole cutaneous feedback is reduced experimentally through cooling or anaesthesia (Perry et al. 2000, 2001; Nurse and Nigg 2001; Eils et al. 2002; Meyer et al. 2004) impairments in postural control are observed. Additionally, enhancement of foot sole cutaneous feedback through applied vibration leads to alterations and illusions of whole body sway and reduced gait variability (Kavounoudias et al. 1999; Roll et al. 2002; Galica et al. 2009). There is a growing interest in investigating strategies to improve postural control through cutaneous feedback augmentation. Facilitatory shoe insoles that employ subthreshold (Priplata et al. 2003, 2005; Galica et al. 2009) and suprathreshold vibration (Novak and Novak 2006), as well as static rigid support (Perry et al. 2008) have been shown to improve balance and gait parameters in older adults, and in patients with diabetes, stroke, and Parkinsons. Although it is well established that foot sole mechanoreceptors play an important role in the control of gait and standing balance, the contributions of individual afferent classes across foot sole locations remain less clear.

Four classes of low-threshold cutaneous mechanoreceptors have been identified in the glabrous skin covering soles of the feet and palms of the hands. Each class is sensitive to deformation and motion of the skin, and provide tactile and kinesthetic sensory feedback (Collins 2005; Lowrey et al. 2010). The firing characteristics of each class is related to the morphology and location of their associated receptor endings within the skin (Johnson 2001). As such, they are inevitably influenced by the mechanical characteristics of the skin. Cutaneous afferents are classified based on their receptive field size (small, Type I and large, Type II) and their ability to adapt to sustained indentation (slowly, SA and fast, FA). Additionally, each afferent class has unique vibration response characteristics. Previous work in the hand has shown that SA afferents are most easily activated by low frequencies; SAII's below 8 Hz and SAI's between 2 Hz and 32 Hz. In contrast, FA are more sensitive to high frequencies, between 8 Hz and 64 Hz for FAI and between 64 Hz and 400 Hz for FAII afferents (Johansson et al. 1982). FAI and FAII afferents provide velocity and vibration feedback (Macefield et al. 1990). In the foot sole, this feedback is important in signaling step breaking and propulsion as well as responding to slips and trips. SAI afferents transmit information about the magnitude and rate of pressure applied to the skin (Macefield et al. 1990), while SAII afferents signal stretch and can respond to movement of the joints, including the ankle (Aimonetti et al. 2007). The SAII afferents are relatively insensitive to indentations and vibrations normal to the skin, and as such vibration perception threshold (VPT) testing is thought to target the SAI (<5 Hz), FAI (8–60 Hz) and FAII afferents (>60 Hz) (Löfvenberg and Johansson 1984; Bolanowski et al. 1988).

Tactile feedback from the hands and feet purportedly arise from the same receptors yet there are notable differences in receptor distribution and firing characteristics between these regions (Kekoni et al. 1989; Kennedy and Inglis 2002). In the finger tips, increased mechanoreceptor density corresponds to higher tactile sensitivity compared to the less densely innervated palm (Löfvenberg and Johansson 1984; Vallbo and Johansson 1984). High afferent density increases the likelihood of a stimulus to activate a perceptually meaningful response in one, or a population of afferents. In contrast, the current literature supports an even distribution of mechanoreceptors across the foot sole despite regional differences in tactile sensitivity (Kekoni et al. 1989; Kennedy and Inglis 2002; Hennig and Sterzing 2009). Additionally, cutaneous afferent firing thresholds are higher and receptive field size larger in the foot sole compared to the hand (Johansson and Vallbo 1980; Kennedy and Inglis 2002). These studies indicate that foot sole cutaneous afferents are less sensitive than the hand, and regional sensitivity differences in the foot sole cannot be accounted for by differences in mechanoreceptor density. It has been suggested that regional afferent firing and perceptual threshold differences between the hands and feet could reflect differences in the mechanical properties of the skin (Kekoni et al. 1989; Kowalzik et al. 1996; Kennedy and Inglis 2002).

The functional role of the foot sole subjects it to high mechanical pressures and shear forces (Tappin and Robertson 1991; Hayafune et al. 1999). In response to repetitive load application, there is a local thickening of the skin due to accelerated keratinization as well as increases in the number and diameter of collagen fibers (Wang and Sanders 2003; Kim et al. 2010). Callus formation allows the skin to withstand greater stresses but at what sensory cost? Skin exhibits nonlinear viscous properties, and consequently, the transmission of tactile stimuli through the skin is velocity and frequency dependent (Wu et al. 2006). The relationship between the mechanical properties of the skin and tactile sensitivity across the foot sole remain unclear.

The purpose of this study was to make the first direct comparison between perceptual threshold across the foot sole and skin hardness, epidermal thickness, and stretch response. The aim is to understand the relationship between tactile perception and the mechanical properties of the glabrous skin on the foot sole. There is expected to be a positive relationship between skin hardness, thickness, and stretch response with increased tactile threshold (decreased sensitivity), which will account for regional sensitivity differences.

## Materials and Methods

### Participants

Twenty-two volunteers recruited from the University of Guelph and the Canadian Memorial Chiropractic College (CMCC) (10 male, 12 female, age 18–31 mean 24) participated in this study. Each participant was tested in the same temperature controlled laboratory at CMCC. All subjects were free of peripheral neuropathy and reported no other known neurological conditions. Following an explanation of the protocol, each subject gave written, informed consent to participate in the experiment, which was approved by the University of Guelph and the CMCC research ethics boards and is in agreement with the declaration of Helsinki.

### Perception threshold tests

**Figure 1:**
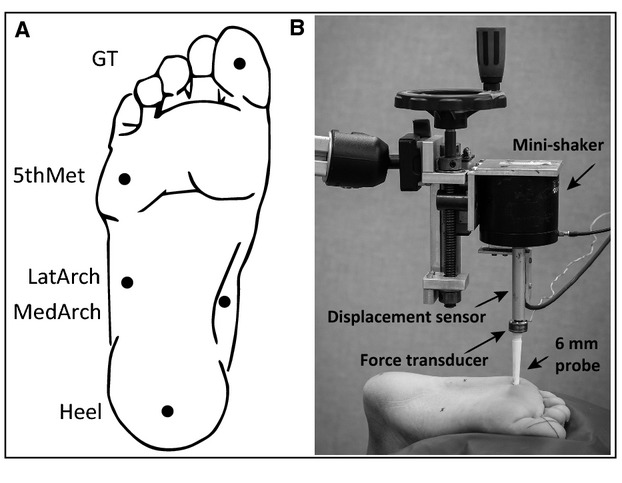
(A) Foot sole test sites. The Heel location was marked 15% anteriorly along the length of the foot. The MedArch and LatArch locations were marked 15% along the width of the center of the arch from the medial and lateral borders, respectively. The 5th Met location was 15% of the length along the metatarsals from the lateral border. The GT location was centered on the pad of the distal phalange. (B) Vibration perception testing set up. Vibratory stimuli (3, 40, 250 Hz) were delivered with a mini-shaker through a 6 mm diameter probe positioned perpendicular to each test site with 2N of preload. Threshold values are reported in micrometers of displacement, which were recorded with a displacement sensor.

Vibration perception threshold (VPT) at three frequencies (3, 40, 250 Hz), as well as monofilament (MF) perception threshold, was determined across five foot sole locations on the right foot. Test sites included the great toe (GT), fifth metatarsal head (5th Met), lateral arch (LatArch), medial arch (MedArch), and heel (Heel) (Fig.1A). Test sites were standardized to a percentage of foot sole length and width; measurements were taken of foot length, from the back of the mid-heel to the second toe, and width across the metatarsals and arch. For VPT testing, participants lay prone with their right knee flexed (90°) and leg supported in a brace; the leg was extended and ankle supported for MF testing.

The vibration stimuli were delivered using a Vibration Exciter (Mini-shaker type 4810, Bruel & Kjaer, Naerum, Denmark, 6 mm diameter probe) secured in a custom holder (Fig.1B). The probe was positioned perpendicular to the test site and a force transducer (load cell model 31, Honeywell, MN) was placed in series with the probe to control the preload (2N) for all trials. A displacement sensor (model RGH24Z, Renishaw, Glouscestershire, U.K.) digitized the peak-to-peak displacement of the probe (1000 Hz, 0.5 *μ*m resolution). VPT was measured at three different frequencies (3, 40, 250 Hz) using a binary search method (Perry 2006). Three trials, of 11 iterations (iteration is a 2-sec vibratory burst followed by a 3–5 sec pause), were presented at each frequency/site. The first iteration of each trial always consisted of a suprathreshold stimulus while the second iteration was subthreshold. Subjects were instructed to press a trigger as soon as they could detect the vibration. Pressing the trigger within the 2-sec window resulted in a ‘true’ response while ‘false’ responses occurred when the stimuli were not perceived and/or the trigger not pressed (within the 2-sec time frame). A true response resulted in a decrease in magnitude by half of the previous true response, while a false response resulted in an increase in magnitude halfway between the last false and true responses. The smallest perceived displacement (*μ*m) over the 11 iterations for each of the three trials was averaged to give the VPT at each frequency/site. All three frequencies were tested at one site before moving onto the next. The order of sites and frequencies tested were randomized across subjects.

Monofilament threshold was assessed using Semmes-Weinstein monofilaments (North Coast Medical Inc, Gilroy, CA). Site order was randomized, and the same experimenter applied the monofilaments for each subject, using a modified 4-2-1 search method (Dyck et al. 1993). Participants were instructed to be at least 90% confident in their responses, and were informed that multiple catch trials would be presented in which no monofilament would be applied. A 3-2-1 countdown was given before monofilament application (1.5 sec on, 1.5 sec removal), to which a ‘yes’, or ‘no’ response was required. Threshold was determined to be the lowest monofilament (grams of force) correctly perceived at least 75% of the time. Across subjects, MF threshold was reached at each site after an average of 12 trials (range 9–16) with 1–2 catch trials for each site. Number of trials was based on positive and negative response ratio to establish threshold level. Two tests were performed at each site and averaged.

### Mechanical property measurements

Skin temperature, hardness, epidermal thickness, and stretch response measurements were taken at each test site.

Temperature was measured with an infrared digital thermometer (THS841-065 Combo Thermometer, ThermoWorks, Orem, UT) prior to the VPT testing to confirm that skin temperature was within a normal range (Sun et al. 2005).

Hardness measurements were taken using a handheld durometer (Type 1600-OO, Rex Gauge, Brampton, ON, Canada) with a 2 mm diameter, column-shaped indenter. This style of durometer is ideally suited for taking skin hardness measurements (Kissin et al. 2006) and have shown excellent repeatability across the foot sole (Cuaderes et al. 2009). Durometers determine hardness by measuring the penetration of an indenter into the skin, which gives a reading of increasing hardness from 1 to 100 (arbitrary units, au). To assess creep, a reading was taken within the first second of contact, followed by a second reading 10 sec after sustained application. This was done twice per site. There were no significant differences between the initial and final durometer readings (*P* = 0.658); therefore all four measurements were averaged to give a single measure of hardness (au) at each site.

**Figure 2:**
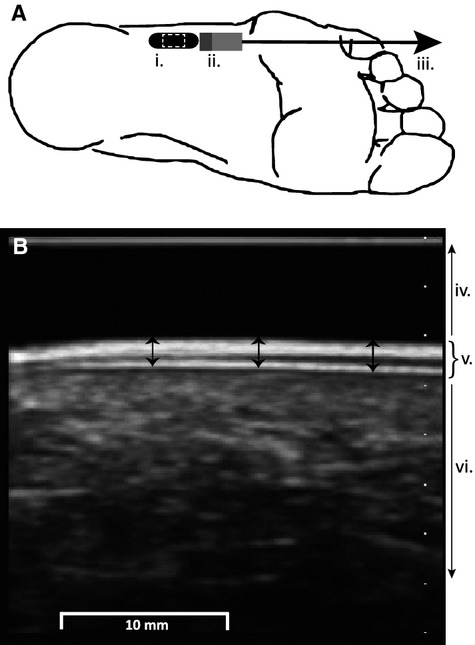
(A) Ultrasound and skin stretch set up. Ultrasound transducer location (i.) for the LatArch test site, with the image site outlined by a white dashed line. A plastic tab (ii.) glued to the skin facilitated 10 mm of pull (iii.). (B) B-mode ultrasound image including the standoff pad (iv.), epidermis (v.) and dermis plus subcutaneous tissue (vi.). Three measurements of epidermal thickness (arrows) were taken and averaged at each test site.

Epidermal thickness (measured to the nearest 0.01 mm) and stretch response (pseudo-stiffness; maximum cumulative lateral displacement of the epidermis expressed as a % of a 10 mm applied pull) were obtained using high-frequency (40 MHz) B-mode ultrasound (Ultrasonix RP, Burnaby BC). Images were taken with an L14-5/38 ultrasound transducer sectored to 50%, resulting in a 19 mm wide image, centered over the test site in line with the long axis of the foot. The transducer was held by hand and positioned on a 5 mm thick agar standoff pad to reduce interference at the transducer contact interface and to optimize the focal zone at the level of the epidermis. Image depth was set to 2 cm with a single focal zone at the level of the epidermis which appears as bilaminar, parallel hyperechoic lines (Fig.2B) (Wortsman 2012). At each site, three epidermal thickness measurements were taken and averaged using Image Tool 3.0 software for Windows (Image Tool version 3.0, The University of Texas, Health Science Center, San Antanio, TX). With the ultrasound transducer held stationary, a 10 mm 8.3 mm/sec anterior pull, parallel to the long axis of the foot was applied to the skin using a MultiTest-i machine (Mecmesin, Sterling, VA). String conveyed the pull from the MultiTest-i through a plastic tab (2 cm wide, 5 cm long) glued 1 cm anterior to each test site (Fig.2A). A consistent preload of 1.9–2.1 N was used to remove any slack in the line prior to each pull. Ultrasound data were processed with a custom program (MATLAB 7.1; The MathWorks Inc., Natick, MA). Displacement of the epidermal tissue was determined from the “raw” ultrasound radio frequency data using cross-correlation techniques (Ophir 1991; Konofagou and Ophir 1998; Langevin et al. 2011) with custom software written in MatLab (Natick, MA). A region of interest (1 × 1.5 cm) was defined within the approximate center of each ultrasound frame (Fig.2B). Motion occurring between successive frames of radio frequency data was accumulated over the duration of the stretch testing to yield a cumulative displacement value. The epidermal stretch response represents the maximum cumulative lateral displacement divided by the applied pull (10 mm) and expressed as a percentage. Epidermal thickness and stretch response measurements were taken after MF, VPT, and hardness testing to avoid any potential effects of skin hydration on perceptual threshold or hardness that may have been caused by the moist agar stand-off pad. Skin hydration has been shown to have a nominal effect on VPT, but does influence spatiotemporal acuity as well as epidermal structure (Warner et al. 2003).

### Ranked data

To further investigate sensitivity and mechanical property relationships across the foot sole, each perceptual threshold test (3, 40, 250 Hz VPT and MF) and the mechanical property measurement (hardness, thickness, stretch response) were ranked across test sites for each subject. For each perceptual threshold test, sites were ranked 1-to-5 with 1 indicating the site with the lowest perceptual threshold, and 5 being the highest perceptual threshold. Likewise, the softest and thinnest sites, as well as sites with the smallest stretch responses were given a rank of 1, while the hardest, thickest and sites with the largest stretch response were given a rank of 5. The ranks for the perceptual threshold tests and mechanical property measurements were averaged across subjects to highlight the site order relationship for these measures.

### Analysis

Outliers, defined as a large deviation from the mean (±3SD), were removed from the data set (VPT: 3 of 330 data points, and MF: 4 of 110 data points). Technical issues during data collection resulted in an additional 10 missing VPT data points. Residuals were tested for normality (Shaprio–Wilk) and homogeneity of variance (Brown and Forsythe) and data were corrected with a log transformation when necessary. One-way repeated-measures analysis of variance (ANOVA) with post hoc comparisons (Tukey–Kramer) was used to compare foot sole perceptual threshold and mechanical property measures across foot sole sites. To account for between subject variability, data were normalized to subject mean foot sole values (site thresholds, thickness, hardness were expressed as a percentage greater or less than the mean). Linear regression (Pearson's product) analysis was used to calculate the coefficient of determination (*R*^2^) between foot sole site perceptual thresholds (VPTs and MF) and mechanical properties (skin hardness, epidermal thickness and stretch response) for both raw and normalized values across all subjects. Individual subject correlations were further examined to determine the direction and strength of subject correlations within the population (Fig.6). Ranked perceptual threshold and mechanical property measurements were evaluated for foot sole site differences using a Kruskal–Wallis test and Dunn's post hoc analysis for nonparametric data. SAS statistical software version 9.3 (SAS Institute, Cary, NC) was used for all parametric statistical analysis and Prism5 was used for nonparametric analysis (GraphPad Prism version 5.0c for Mac OS X, San Diego, CA). For all tests, significance was determined at a type-I error rate of *P* < 0.05.

## Results

Average foot sole temperature was 25.6°C with a range of 23.4–28.6°C, which is normal for this population (Sun et al. 2005). There were no sex differences present for any perception threshold or mechanical property tests (*P* > 0.05). Male and female subjects were combined for data analysis.

### Perception threshold tests

**Figure 3:**
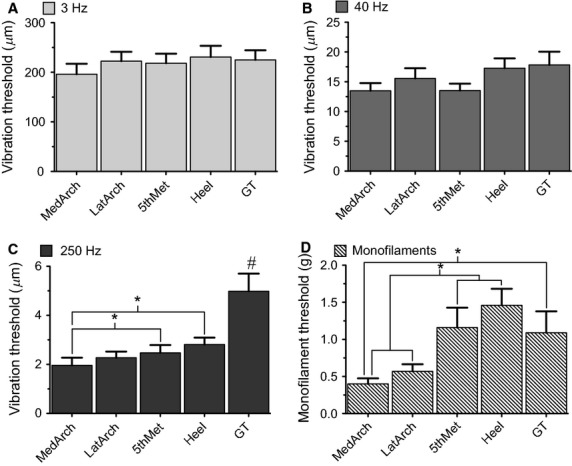
Mean vibration and monofilament perception thresholds across the foot sole with standard error. No significant differences were found across site at 3 Hz (A) or 40 Hz (B) VPT. Significant differences were found at 250 Hz VPT (C) and MF (D). *#* indicates a significantly higher threshold of the GT compared to all other sites and * denotes a significant difference (*P* < 0.05).

Vibration perception threshold significantly decreased (sensitivity increased) with increasing test frequency (*P* < 0.0001). Hence, the ability to detect a vibration was significantly greater at 250 Hz (2.90 *μ*m) than at 40 Hz (15.54 *μ*m) and greater at 40 Hz compared to 3 Hz (218.51 *μ*m), across all foot sole sites. A significant main effect of site was found for VPT, but only for vibration at the 250 Hz frequency (*P* < 0.0001) (Fig.3). Significant site differences were also found for MF testing (see below). Post-hoc analyses indicated that the GT had the highest vibration threshold at 250 Hz (4.98 *μ*m); significantly higher than all other sites across the foot sole (*P* < 0.0001), while the heel was the second least sensitive at 250 Hz with a threshold of 2.81 *μ*m. The MedArch had significantly lower vibration threshold at 250 Hz (1.96 *μ*m) compared to all sites (*P* < 0.05) except for the LatArch (*P* = 0.068). At 250 Hz, the MedArch was found to be the most sensitive site in 58% of subjects, while the GT and Heel were never the most sensitive. Neither of the lower frequencies, 3 Hz or 40 Hz, demonstrated significant site differences for VPT.

For MF testing, the Heel had the highest threshold (1.46 g), followed by the 5th Met (1.16 g) and GT (1.09 g). Following statistical analysis, the Heel and 5th Met were shown to have significantly higher MF thresholds compared to both the LatArch (0.57 g) and the MedArch (0.40 g, *P* < 0.0001). The GT MF threshold was also significantly higher than the MedArch (*P* = 0.0002, Fig.3D). The relationship between perception threshold and test site is presented in Table1.

**Table 1:** Foot sole test site mechanical properties and perceptual threshold rankings with mean test values.

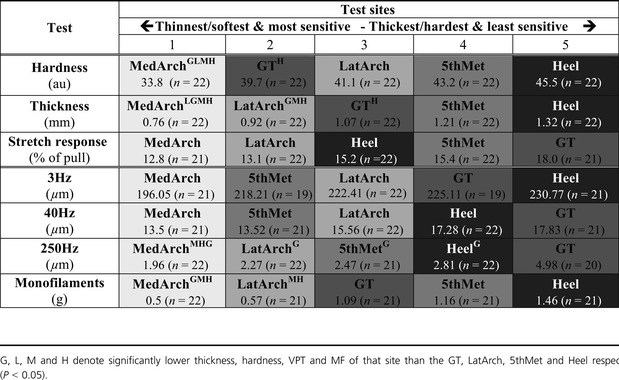

### Mechanical property measurements

**Figure 4:**
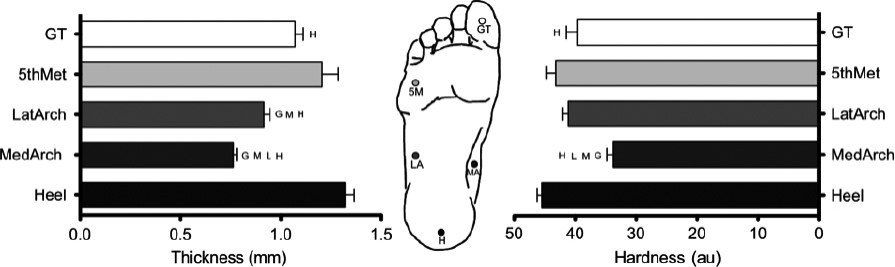
Mean skin thickness and hardness across the foot sole with standard error. Circles represent the test site. G, M, L and H denote significantly lower mechanical property measures than the GT, 5th Met, LatArch, and Heel, respectively (*P* < 0.05).

Skin hardness and epidermal thickness showed significant site differences (*P* < 0.05) (Fig.4), while there were no sites differences in epidermal stretch response (*P* = 0.31) (Table1). The Heel was both the hardest (mean 45.5 au, range 38.5–51.5 au) and thickest (mean 1.32 mm, range 0.90–1.70 mm) site, while the MedArch was the softest (mean 33.8au range 22.0–40.3 au) and thinnest (mean 0.76 mm range 0.62–0.90 mm). The 5th Met, GT and LatArch were found to have intermediate hardness and thickness. Skin mechanical property measurements for all sites are presented in Table1.

### Correlations

**Figure 5:**
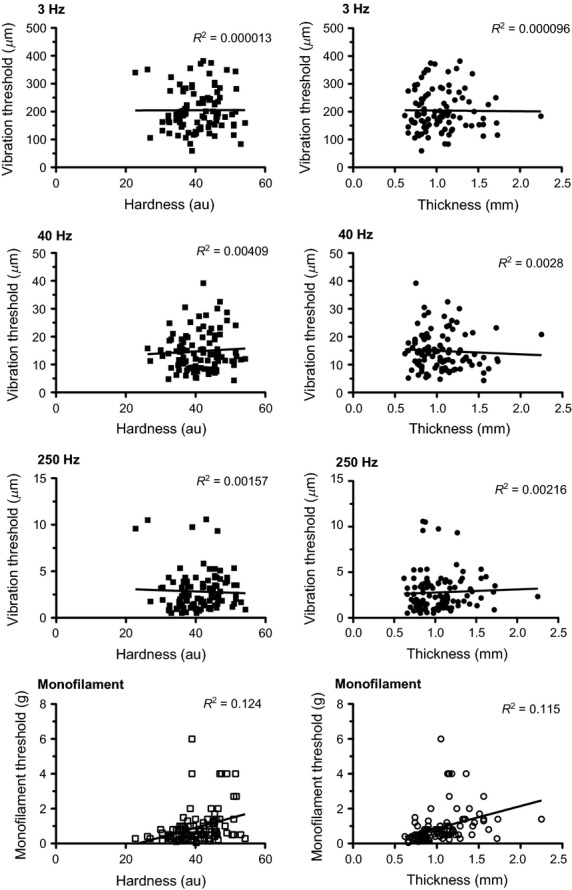
Correlations between perception threshold and mechanical property measurements. There were no significant relationships between site vibration or monofilament perception threshold with skin harness and site thickness (*P* > 0.05).

**Figure 6:**
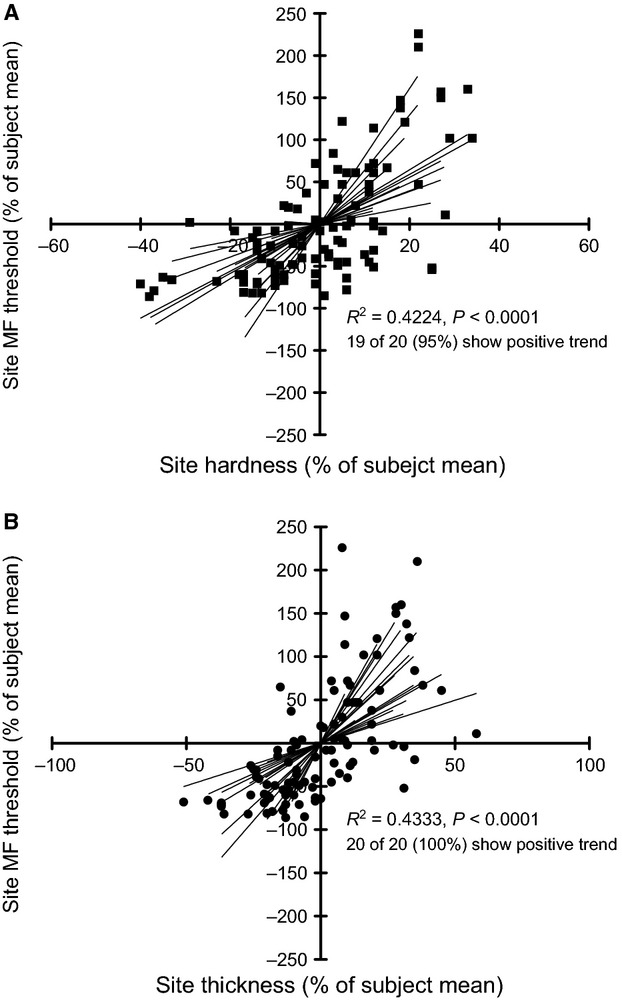
Normalized monofilament threshold compared to (A) hardness and (B) thickness. Values are plotted as percentages greater (positive) or less (negative) than subject mean values. Trend lines are plotted for individual subjects, which show a majority of positive correlations. Combined, significant linear correlations were found between normalized MF and hardness and thickness.

Hardness and thickness showed a positive correlation to each other (*R*^2^ = 0.8327 *P* = 0.0307) while neither were significantly correlated with stretch response (hardness *R*^2^ = 0.3736 *P* = 0.5357, thickness *R*^2^ = 0.5798 *P* = 0.3056). Correlations between (non-normalized) foot sole sensitivity and mechanical property measurements did not reveal any significant relationships (Fig.5). Normalized MF thresholds did, however, show moderate positive correlations with hardness (*R*^2^ = 0.4224, *P* =< 0.0001) and thickness (*R*^2^ = 0.4333, *P* < 0.0001) (Fig.6). Out of the 20 subjects, 19 had positive correlations between MF threshold and hardness (40% with an r > 0.7) and 20 of 20 had positive correlations with thickness (60% with an *r* > 0.7). Normalized VPT did not correlate with normalized mechanical properties.

### Ranked responses across sites

There was sizable variability in vibration perception thresholds both between and within subjects. After outliers were removed (±3SD, VPT 3 of 330 data points), VPT range remained large, with max vibration thresholds calculated as 5, 6 and 9 times larger than the minimum values for 3 Hz, 40 Hz, and 250 Hz VPT, respectively. In contrast, the ranges for thickness (0.76–1.32 mm) and hardness (33.8–45.5au) across sites were relatively small. As such, a relationship to site mechanical properties may have become lost due to minimal room for variation of the dependent variables hardness and thickness.

**Figure 7:**
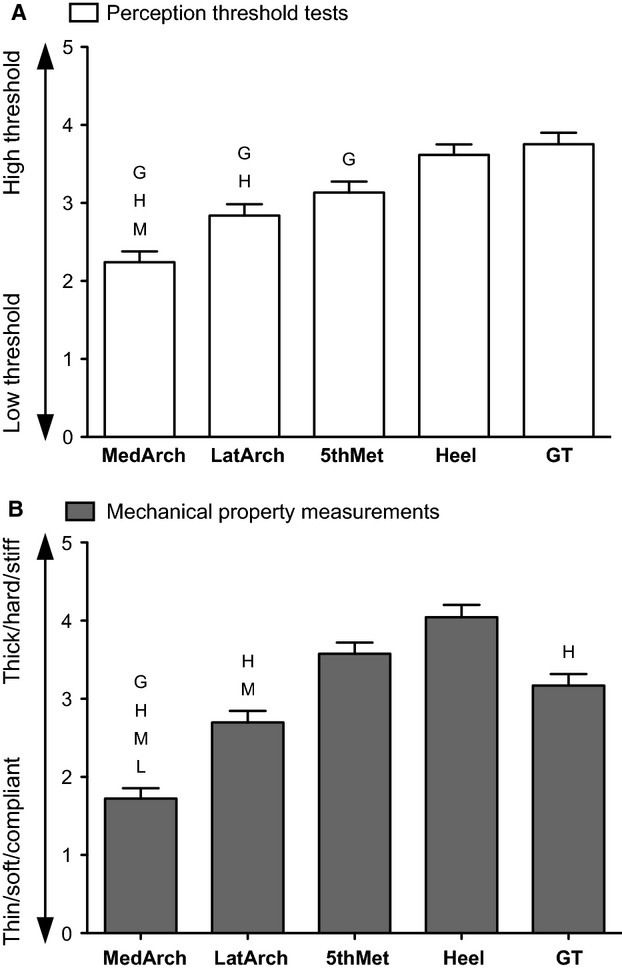
Foot sole sites ranked by perceptual threshold (A) and mechanical properties (B). For each subject, a rank of 1 was given to the site with the lowest, and a rank of 5 to the site with the highest perceptual threshold or mechanical property measurement. Ranks were averaged across subejects for each foot sole site. Error bars represent the standard error of the mean. L, M, H and G denotes a significantly lower ranking of that site than the LatArch, 5th Met, Heel, and GT, respectively (*P* < 0.05).

As an attempt to standardize the perceptual responses and mechanical changes across the foot sole, data were ranked. Ranked perceptual threshold tests and mechanical property measurements show a similar relationship across the foot sole (Fig.7). Perceptually, the MedArch had significantly lower ranked thresholds compared to all sites except for the LatArch (*P* < 0.05). The MedArch was also the lowest ranked site for mechanical properties (*P* < 0.05). In contrast, the GT and Heel were the two highest ranked sites for perceptual thresholds (*P* < 0.05) and the Heel was ranked highest for mechanical properties compared to all sites (except for the 5th Met (*P* < 0.05). The GT was ranked in the middle, significantly higher than the MedArch but lower than the Heel (*P* < 0.05). The ranked responses highlighted that with the exception of the GT, sites with low perceptual thresholds always had relatively low mechanical property values.

## Discussion

The aim of this study was to investigate if differences in skin sensitivity across the foot sole are influenced by variations in the mechanical properties of the skin, as has been suggested in the literature (Kekoni et al. 1989; Kowalzik et al. 1996; Kennedy and Inglis 2002). Skin hardness, epidermal thickness, and stretch response were directly compared to vibration and monofilament perception thresholds across five-foot sole sites. Sites that were harder, thicker, and stiffer were expected to be less sensitive compared to softer, thinner and more compliant sites, and perceptual thresholds were expected to show a positive linear relationship with mechanical property measurements. Ranking foot sole sites on sensitivity and mechanical property measurements demonstrated that in most instances, sites that were relatively sensitive also had softer and thinner skin compared to less sensitive sites. In following with previous literature, the MedArch was found to be the most sensitive site, while the Heel was the least sensitive with the exception of the GT at 40 Hz and 250 Hz (Kekoni et al. 1989; Nurse and Nigg 1999). The MedArch was also the softest and thinnest site, while the Heel was the hardest and the thickest. Despite this trend, the results were unable to establish causality between the mechanical properties of the skin and vibration and MF sensitivity. It appears that small differences in foot sole skin mechanics observed in young healthy adults do not have an observable influence on vibration sensitivity, and only a minimal influence on MF sensitivity. When normalized to subject mean values, MF thresholds did show positive correlations with normalized hardness and thickness suggesting that increases in skin hardness and epidermal thickness may elevate MF threshold. These data suggest that the mechanical properties of the skin could have a minimal, yet significant, influence on the ability to perceive light touch through MF testing, however larger ranges in hardness and thickness are likely required to evoke a meaningful change in vibration sensitivity.

### Skin mechanical properties influence stimulus transmission

Mechanical properties of the skin have frequently been proposed to account for sensitivity differences between the glabrous skin on the foot and hand and between different sites across the foot sole (Kekoni et al. 1989; Kowalzik et al. 1996; Kennedy and Inglis 2002). To date, the majority of studies have used computer and animal models to address the influence of skin mechanics on afferent and perceptual thresholds. Using a biomechanical fingertip model, the mechanical properties of the skin have been shown to influence the transmission of mechanical vibrations to the underlying mechanoreceptors (Wu et al. 2006). Low frequency vibrations (<31.5 Hz) were shown to induce dynamic strains most effectively in superficial skin layers, while higher frequencies (63–250 Hz) penetrate deeper to depths where the FAII receptor endings reside (Wu et al. 2006). Additionally, a complex ratio of force to velocity has been used in both animal and human models to describe mechanical impedance (resistance to indentation) of the skin and its relationship to stimulus transmission. The variance in stimulus transmission has been suggested to account for differences in FAI afferent firing thresholds in the rat hind paw (Devecıoğlu and Güçlü 2013), and for the differences in sensitivity thresholds at 40 Hz across the human fingertip (Güçlü and Bolanowski 2005). In the current study, while we have not measured cutaneous afferent responses directly, our observations of reduced MF sensitivity are thought to relate to a reduced ability to activate primary afferents (through reduced stimulus transmission) at harder and thicker sites. Increased epidermal thickness creates a greater separation between the mechanoreceptors and external stimuli, which may have a meaningful impact on afferent firing at perceptual threshold. Additionally, skin hardness and stretch response can influence the way skin deforms in response to indentation and stretch stimuli (Takei et al. 2004; Staloff and Rafailovitch 2008). Ultimately the ability to transmit force to activate mechanoreceptors may be affected by skin hardness and thickness, and the ability to activate the mechanoreceptors is essential to evoke afferent firing and to create a percept.

To further examine the mechanical characteristics of the skin, the epidermal stretch response was investigated. This measure provided a novel pseudo-stiffness variable to better understand the shearing forces exhibited at the epidermal–dermal interface at different foot sole sites. During gait, the acceleration and deceleration phases subject the foot sole to large shearing forces (Tappin and Robertson 1991). The ability of the skin to deform in response to stretch will influence afferent firing, notably SAII afferents which are particularly sensitive to skin stretch (Kennedy and Inglis 2002). Counter to the hypotheses, and unlike skin hardness and thickness, no significant site differences were observed for stretch, and stretch response measures did not correlate with any perceptual threshold tests. The lack of correlation between stretch response and perceptual threshold is attributed to the small range of stretch response found across test sites (1.28–1.80 mm) and high variability (standard deviation 1.29 mm). Perhaps more importantly, however, SAII afferents, whose firing is most greatly influenced by lateral skin stretch, are least sensitive to the perpendicular MF stimuli investigated in the current study (Johansson et al. 1982). As such, a measurable change in the stretch response across sites (if present), while it will differentially activate SAII afferent firing, is unlikely to correlate well to SAII threshold response to MF or vibration.

### Skin mechanical properties influence MF perception

We believe that skin hardness and epidermal thickness contribute to the significant site MF threshold differences in the present study, as a result of altered stimulus transmission to, and subsequent activation of the underlying mechanoreceptors. The influence of skin mechanics on tactile perception appears to be dependent on the type of sensory test used and the tactile afferent population targeted. For example, increased foot sole hardness in diabetic patients as well as in healthy controls corresponded to increased monofilament thresholds across the foot sole (Thomas et al. 2003). While in two additional studies by subsequent authors, foot sole skin thickness did not influence two point discrimination (Kowalzik et al. 1996) and was not able to explain site differences in the perception of electrical stimulation and afferent electrical activation thresholds (Frahm et al. 2013). The results of the latter two studies do not follow a similar causal trend to our work, which may not be surprising given that spatial information (two-point discrimination) and electrical stimulation thresholds are perceptually different from light touch (MF). Two point discrimination is suggested to be mediated by SAI afferents, and electrical stimulation targets A-delta nociceptive fibers, both of which are thought to not be significantly influenced by skin mechanics (Kowalzik et al. 1996; Craig 1999; Frahm et al. 2013). In contrast, MF threshold is mediated by baseline activity of FAI afferents (Johansson and Vallbo 1979a). Our present data suggest that the influence of skin mechanics on altering perceptual threshold in young healthy adults is limited to MF stimuli, which are known to target activation of FA afferents.

### Skin mechanical properties do not influence VPT

Contrary to the hypothesis, there was no significant correlation between thicker, harder and stiffer foot sole sites and elevated VPT thresholds across any frequencies tested. Similar to MF thresholds, 250 Hz VPT were found to be significantly different across the foot sole; however unlike MF threshold, correlations of VPT with skin thickness, hardness and stretch response did not reveal any relationships. This is thought to reflect the small range of mechanical property measurements observed in the present study. These small ranges are expected to result in afferent firing patterns that do not differ across the foot sole, which would conceal any relationship between mechanical properties and VPT if present. Additionally, in the present study, VPT testing involved 2N of preload combined with a 6 mm diameter probe. This may have led to both SA afferent adaptation and increased spatial summation (greater afferent contribution) at the contact site, which could have masked any influence of skin mechanics on VPT. Differences in contact area, pre-load, stimulus quality and subject expectations also make comparisons between MF and VPT difficult. In contrast to VPT, MF thresholds reflect minimal activity in (potentially just single) FAI afferents where subtle differences in firing threshold (because of few afferents) may have a meaningful influence on perception (Johansson and Vallbo 1979a). Due to the large baseline of firing with VPT (6 mm probe), a greater absolute change in afferent firing necessitates greater differences in skin mechanics to alter VPT compared to MF thresholds across the foot sole.

### The role of cutaneous afferent classes in mediating perceptual threshold

MF and vibration perception threshold is set by the capability of the most sensitive afferents to provide a meaningful response (percept). In this way, vibration perception threshold testing is thought to allow the sensory contributions of the different cutaneous afferent classes (FAI, FAII, SAI, SAII) to be selectively investigated (Johansson et al. 1982; Bolanowski et al. 1988; Kekoni et al. 1989). While reported in the hand (Johansson et al. 1982), individual cutaneous afferent vibration tuning curves have not been established in the foot sole. As a consequence, afferent firing thresholds at different frequencies across the foot sole are not well understood. Based on the hand literature, the test frequencies in the present study are thought to target the SAI (3 Hz), FAI (40 Hz) and FAII (250 Hz) afferents, however overlap in afferent class firing is expected (Löfvenberg and Johansson 1984; Bolanowski et al. 1988). In the current work we found significant differences in 250 Hz VPT across foot sole sites, which are believed to be attributed to FAII firing. 250 Hz VPT amplitudes are small (mean 2.9 *μ*m), and therefore a change in threshold of 1 *μ*m across foot sole sites amounts to a 34% increase or decrease, and subsequently a significant perceptual difference. In contrast, a 1 *μ*m difference in amplitude at 3 Hz and 40 Hz VPT is only a 0.5% and 6% change in VPT, respectively. The lack of site differences for 3 Hz and 40 Hz VPT found in the present study indicates that small changes in SAI and FAI afferent firing across sites is not able to evoke detectable changes in perception due to the inherently large threshold amplitudes at 3 Hz (218 *μ*m) and 40 Hz (15.6 *μ*m). Although it is not clear how cutaneous afferent firing varies across the foot sole in density and sensitivity, the frequency specific VPT differences in the present study are in agreement with previous work which found high frequency VPT to show more regional differences across the foot sole compared to low frequencies (Kekoni et al. 1989; Nurse and Nigg 1999). The present data do not support that these regional differences at 250 HZ VPT across foot sole sites are due to hardness and thickness.

Monofilaments measure light-touch threshold, which is suggested to reflect the activity of a small number, or perhaps even single, FAI afferents (Johansson and Vallbo 1979a). The current study found MF threshold to be significantly different across the foot sole, and when normalized to individual mean values, to show a moderate, positive correlation with skin hardness and epidermal thickness. This supports a potential influence of skin mechanics on FAI firing threshold at perceptual threshold levels. Micro-stimulation studies have shown that activity in single FAI afferents can lead to meaningful percepts (Ochoa and Torebjörk 1983; Macefield et al. 1990). In some cases single impulses from FAI afferents innervating the fingertips were detected as taps. In contrast electrical stimulation of FAII afferents, required temporal summation and stimulation frequencies >10 Hz to evoke sensations of vibration in the hand (Ochoa and Torebjörk 1983). Monofilaments apply very light localized pressure, and threshold stimuli require only minimal afferent spatial or temporal summation to evoke a percept. In this way, MF testing may provide information, albeit indirect, about FAI afferent sensitivity. Although the relationship between primary afferent firing and skin hardness and thickness in the foot sole have not been investigated directly, reduced local skin deformation in harder sites and increased separation between stimulus and mechanoreceptor in thicker sites is thought to contribute to the observed differences in MF across the foot sole.

### Additional factors that may influence foot sole sensitivity

The mechanical properties of the skin are just one of a number of factors which can impact vibration and light-touch threshold. Afferent density and distribution, central mechanisms as well as the physical dimensions of the stimulation site may all convey some influence on perceptual threshold. In the glabrous skin of the hand, there is an increasing proximal-distal gradient in FAI and SAI afferent density (Johansson and Vallbo 1979b). This corresponds to better spatial acuity (grating orientation discrimination) (Craig 1999) and vibratory perceptual thresholds in the fingertips compared to more proximal locations on the finger and palm (Löfvenberg and Johansson 1984). The current literature however, does not indicate a denser innervation of afferents in the arches compared to the Heel or GT and therefore cannot explain the observed sensitivity differences across the foot sole (Kennedy and Inglis 2002; Fallon et al. 2005; Lowrey et al. 2013). Moreover, afferent density gradients, when present, may not be important in all aspect of tactile sensitivity. Monofilament and light-touch perception thresholds are not influenced by afferent spatial summation and increased afferent density would have little influence in these tests (Johansson and Vallbo 1979a). Interestingly, in the Johansson and Vallbo (1979a) study, primary afferent firing thresholds in response to light touch did not differ between the fingertips and palm; which lead the authors to suggest that tactile feedback arising from the fingertips could be deemed more significant by the CNS, and is therefore weighted more heavily centrally, leading to lower perception thresholds. The same case is not as strong in the foot sole where location specific cutaneous feedback demands are less obvious. A comparison between afferent firing and perceptual thresholds across the foot sole has not been done and central mechanisms, which may help explain potential afferent-perceptual threshold discrepancies, are not clear. This does not rule out the potential of the CNS to favour feedback from different foot sole sites, however future work is needed to clarify the degree of cutaneous afferent and perceptual sensitivity variability across the foot sole to better understand additional central and peripheral factors.

### The great toe

The GT was unique compared to the other foot sole sites in that it demonstrated the highest 250 Hz VPT despite having only moderate thickness and hardness measurements. The shape of the GT creates a confined surface area, and has a limited potential for spatial summation. This is thought to result in relatively less afferent activation for a given stimulus compared to the other test sites. This is particularly relevant at 250 Hz VPT as spatial summation is known to play a role in high frequency perception, mediated by FAII afferents. Increasing probe size has been shown to reduce high frequency VPT, but not at lower frequencies (Bolanowski et al. 1988; Kekoni et al. 1989; Gu and Griffin 2013). In the current study, it is believed that afferent summation constraints as a consequence of the physical dimensions of the GT, played a larger role in dictating 250 Hz VPT than skin hardness or epidermal thickness compared to the other foot sole sites. The inclusion of information from additional toes, with different sizes, may help determine the impact of physical constraints on VPT in future studies.

### Ordered relationship between perceptual threshold and mechanical properties

When foot sites for each subject are ranked for sensitivity and mechanical property measurements, it is evident that sites with the smallest mechanical property measurements (soft, thin, compliant) were most often the most sensitive sites. Ranking permits each perception and mechanical property test to be grouped, which provides a broad look at the relationship between sensitivity and mechanical properties across the foot sole. The MedArch consistently had the lowest perceptual thresholds while being the softest, thinnest and most compliant site. In contrast the Heel had relatively high perceptual thresholds paired with relatively large hardness, thickness and stretch response measurements compared to the other test sites. Although between-site differences in foot sole sensitivity and mechanical properties may be small and variable, the ranked data reveal that the order of these measurements is generally conserved. Ultimately, our data show that this relationship is more complex at the individual level and is heavily influenced by factors other than skin mechanics.

### Limitations

The experimental procedures used in the presented study warrant mention of potential limitations in the interpretation of the results. Most notably, the resolution of the displacement sensor (0.5 *μ*m) was close to 250 Hz VPT levels. As a result 250 Hz VPT values <0.5 *μ*m reflect an average of three trials and not actual measurement resolution. The displacement sensor was, however, shown to be reliable and calibrated in 0.5 *μ*m steps. In addition, because the vibration probe size was constant and static surrounds and masking stimuli were not employed, the interpretation of specific afferent class contributions to VPT is limited. Future work is needed to create vibration tuning curves for afferent classes to in the foot sole to strengthen such comparisons.

## Conclusions

In summary, skin mechanics seem to play a role in tactile sensitivity across the foot sole, however this relationship may only be meaningful at perceptual threshold levels, when targeting specific afferent classes (FAI). For a young healthy population, mechanical properties (hardness, thickness, and stiffness) of foot sole skin were not found to have a measureable influence on vibration perception threshold. This novel finding reveals that our VPT differences across foot sole sites, which are in agreement with previous work, cannot be accounted for by variability in skin mechanics. In addition, the current work supports a minimal, yet significant influence of skin hardness and thickness on the ability to perceive light touch through MF testing. We believe that the MF foot sole sensitivity differences found across sites represent regional variations in FAI afferent activation caused by the variability in the mechanical properties of the skin. A better understanding of the relationship between primary afferent firing and perceptual thresholds would help to strengthen this conclusion. Such insight along with our current findings will benefit future investigations that link perceptual thresholds with receptor function.
